# Diphenyl Diselenide-Assisted Radical Addition Reaction of Diphenyl Disulfide to Unsaturated Bonds upon Photoirradiation

**DOI:** 10.3390/molecules28062450

**Published:** 2023-03-07

**Authors:** Yuki Yamamoto, Qiqi Chen, Akiya Ogawa

**Affiliations:** 1 Department of Applied Chemistry, Graduate School of Engineering, Osaka Prefecture University, Osaka 599-8531, Japan; 2 Department of Applied Chemistry, Graduate School of Engineering, Osaka Metropolitan University, Osaka 599-8531, Japan

**Keywords:** dichalcogenide, radical addition, radical substitution, homologous heteroatom catalyst, unsaturated compounds

## Abstract

The addition reaction of interelement compounds with heteroatom–heteroatom single bonds to unsaturated bonds under photoirradiation is an important method for the efficient and atom-economical construction of carbon–heteroatom bonds. However, in practice, the desired addition reaction is sometimes unable to proceed as expected due to the low efficiency of the desired addition reactions or the preferential polymerization of unsaturated compounds. In this study, by combining an interelement compound with homologous heteroatom compounds as a catalyst, we succeeded in suppressing the polymerization of the unsaturated compounds and in attaining a highly selective carbon–heteroatom bond formation through the desired addition reaction. In this paper, we have examined in detail whether such a “catalytic radical reaction” proceeds for unsaturated compounds and found that the dithiolation of some unsaturated compounds (i.e., vinylic ethers, styrenes, and isocyanides) could proceed with the assistance of (PhSe)_2_ under light. The developed methods in this study are expected to have strong implications in the fields of radical chemistry, heteroatom chemistry, synthetic organic chemistry, and catalyst chemistry as atom-economical methods for carbon–heteroatom bond formation.

## 1. Introduction

Associated with “catalytic radical reaction”, two main methods are known so far. The first method is a radical chain reaction using a peroxide or azo compound as a radical initiator, which can be regarded as a catalyst [1,2,3,4]. The second method is a radical reaction that proceeds via an electron transfer process from a metal or functional dye used as a catalyst upon irradiation with visible light [5,6,7,8,9,10]. 

In addition to these methods, if typical element radicals can catalyze another radical reaction, it is expected to be “the third” catalytic radical reaction. For this purpose, in this work, we focused on the utilization of interelement compounds bearing a heteroatom–heteroatom single bond. Homolysis of these bonds under photoirradiation or in the presence of radical initiators can generate heteroatom-centered radicals [11,12,13]. Although their characteristic features were attractive and utilized for the construction of functional molecular scaffolds in recent decades, their catalytic use, as described above, has been very limited [14,15,16,17,18,19,20,21,22]. 

**Scheme 1:**
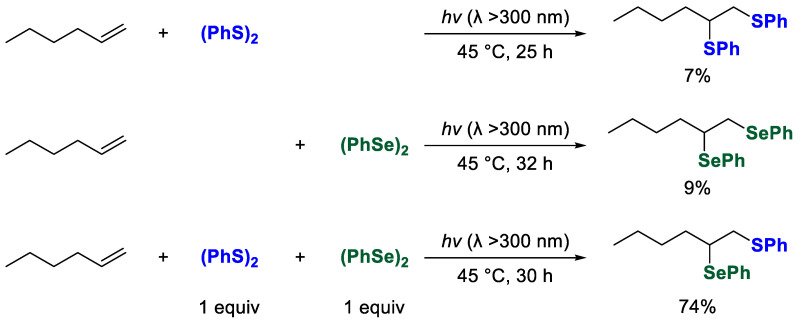
Regioselective thioselenation of alkenes using a (PhS)_2_/(PhSe)_2_-mixed system.

During the course of our investigation, we have previously demonstrated that photoinduced radical addition reactions of interelement compounds to a series of unsaturated compounds proceed efficiently by the combination of interelement compounds [23,24,25,26,27]. In particular, we have succeeded in regioselectively introducing multiple hetero-functional groups into various unsaturated compounds. For example, when group 16 interelement compounds such as organic disulfide or diselenide were used independently, the photoinduced radical addition to alkenes barely proceeded to afford the corresponding adducts in trace yields (Scheme 1). In sharp contrast, the photoinduced radical addition to alkenes proceeds regioselectively when the reaction is carried out by mixing disulfides and diselenides. The results can be explained by referring to the kinetic data of each step in this reaction: The highly reactive PhS• (*k*_PhS•_/*k*_PhSe•_ = ca. 10~50) [28] selectively attacked the terminal position of alkenes, and the thus formed carbon radicals were selectively trapped by (PhSe)_2_, which has a higher carbon radical-trapping ability (*k*_(PhSe)2_/*k*_(PhS)2_ = ca. 160) [29,30]. 

**Scheme 2:**
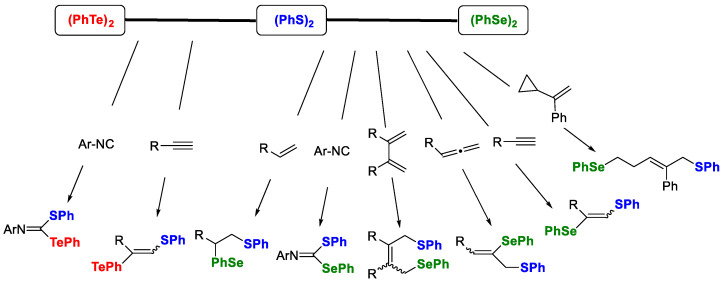
Regioselective introduction of chalcogen functional groups to unsaturated compounds.

The concept of the “heteroatom mixed system” could be applied to a variety of unsaturated compounds such as alkenes, alkynes, allenes, vinylcyclopropanes, and isocyanides, as shown in Scheme 2 [29,31,32,33,34]. In the photoinduced thioselenation reactions, 1,1-addition for isocyanide, 1,2-addition for alkyne and allene, 1,4-addition for conjugate diene, and 1,5-addition for vinylcyclopropane successfully proceeded with excellent regioselectivity. Furthermore, the photoinduced addition to alkyne and isocyanide was also attained by combining (PhS)_2_ and (PhTe)_2_, affording the corresponding thiotelluration products. 

**Scheme 3:**
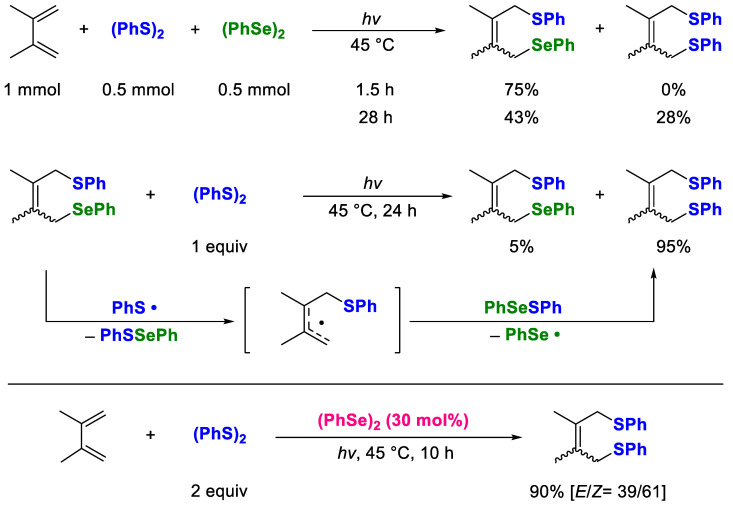
(PhSe)_2_-assisted dithiolation of conjugate dienes with (PhS)_2_ under photoirradiation.

It is known that the reaction of a conjugate diene with (PhS)_2_ causes polymerization of the diene, resulting in a lower yield of the desired 1,4-dithiolation product. Nonetheless, when this reaction was conducted in the presence of 30 mol% (PhSe)_2_ as the additive, the polymerization of the dienes was completely suppressed, and the conjugate addition of (PhS)_2_ to the dienes proceeded efficiently under photoirradiation for 10 h (Scheme 3) [35]. The thioselenation of conjugate diene was complete in about 2 h, and with continued photoirradiation, the thioselenation products gradually transformed into the corresponding dithiolation product because allyl selenide is unstable to near-ultraviolet light. These results strongly suggest the possibility of “the third” catalytic radical reaction. In some cases, we have also investigated these types of replacements with “stoichiometric” reactions; however, the substrate scope and the limitations of the “catalytic” system remain unexplored. 

Hence, in this study, in order to explore the possibility of “the third” catalytic radical reaction, we investigated the multiple introductions of chalcogen-centered functional groups into various unsaturated bonds under radical conditions and further examined in detail whether the selenium and tellurium functional groups of the thioselenation or thiotelluration products could be replaced by sulfur functional groups under radical reaction conditions. We also discussed the possibility of constructing catalytic radical reactions based on the results. 

## 2. Results and Discussion

In the case of organyl selenide, allylic selenides are known to be unstable under photoirradiated (or thermal) conditions because relatively stable allylic radicals can be generated through the cleavage of the allylic C–Se bond. In general, the bond energy of the C–H bond at the allylic position (e.g., CH_2_=CHCH_2_–H, 87 kcal/mol) [36] can be estimated to be about 15 kcal/mol less than that of the C–H bond at the primary alkyl group (e.g., CH_3_CH_2_–H, 101 kcal/mol) [37]. On the other hand, the bond energies of the C–S, C–Se, and C–Te bonds are estimated to be 65.1, 56.0, and 47.8 kcal/mol, respectively [38]. Considering the replacement of C–Se bonds of the thioselenation adducts to C–S bonds under photoirradiation, the following three steps are considered the key factors for a smooth transformation: (1) the cleavage of C–Se bonds under light, (2) the generation of the corresponding relatively stable carbon-centered radicals, and (3) the smooth trapping of the carbon radicals by (PhS)_2_ or PhS–SePh (generated in situ from (PhS)_2_ and (PhSe)_2_). The formed C–S bond has a larger bond energy, and the formed dithiolation adduct might be stable under photoirradiation. However, if the C–Se bond has a large bond energy, it might be difficult to cleave. To achieve the efficient replacement of the C–Se bond with the C–S bond, it is necessary to target not the usual C–Se bond but a C–Se bond activated by some functional groups. On the other hand, the bond energy of the C–Te bond can be estimated to be about 8 kcal/mol less than that of the C–Se bond. It may be possible to replace the C–Te bond with a C–S bond under radical conditions. 

**Scheme 4:**
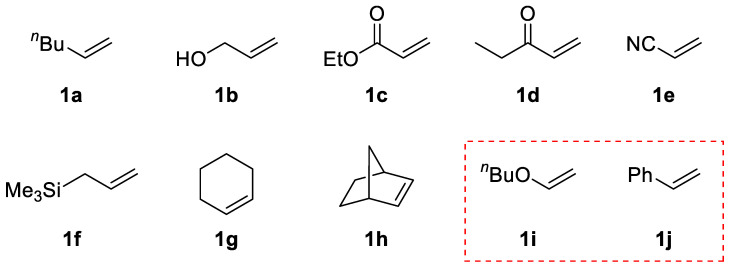
A list of some alkenes for the attempted dithiolation by prolonging the photoirradiation in the thioselenation.

Keeping the difference in the bond energy of C–S, C–Se, and C–Te bonds in mind, we performed thioselenation of some alkenes by prolonging photoirradiation and investigated whether the thioselenation products changed to the corresponding dithiolation products, even if only slightly. The results showed that thioselenation proceeded well for alkenes from **1a** to **1h** in Scheme 4, but no dithiolation adduct was produced as a byproduct. In contrast, thioselenation of butyl vinyl ether **1i** and styrene **1j** resulted in the formation of small amounts of the dithiolation byproducts.

Based on these preliminary experiments, we next focused on vinyl ethers such as 2,3-dihydrofuran to examine bisthiolation assisted by (PhSe)_2_, and the results were compared with those of standard terminal alkenes such as 1-hexene. When a mixture of **1i** (1.0 mmol) and (PhS)_2_ (1.0 mmol) was irradiated for 20 h in the presence of an equimolar amount of (PhSe)_2_, thioselenation product **2** and dithiolation product **3** were obtained in 50% and 10% yields, respectively (Equation (1)). Interestingly, when the photoinduced reaction with 2,3-dihydrofuan **1k** instead of **1i** was conducted under the same conditions, the dithiolation product **4** was obtained as the main product in a 41% yield along with the thioselenation product **5** in a 3% yield, respectively (Equation (2)).

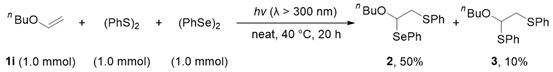
(1)
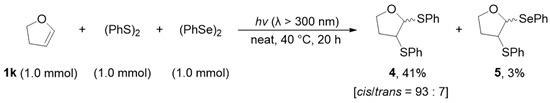
(2)

The results shown in Equations (1) and (2) clearly indicated that the phenylseleno group of the thioselenation products **2** or **5** could be replaced by a phenylthio group under photoirradiation conditions. The results motivated us to investigate the (PhSe)_2_-catalyzed dithiolation of **1k**, and the results are summarized in Table 1. Upon irradiation with a tungsten lamp through Pyrex (*hv* > 300 nm), the reaction of **1k** (1.0 mmol) with (PhS)_2_ (0.5 equiv.) was conducted in the presence of (PhSe)_2_ (30 mol%) for 20 h, yielding 29% of the dithiolation product **4** along with the thioselenation product **5** in a 4% yield (entry 1). Adding 0.1 mL of CDCl_3_ or prolonging the reaction time to 100 h did not improve the yield of **4** (entries 2 and 3). In the latter reaction (entry 3), the yields of the products **4** and **5** decreased, suggesting that **4** and **5** are unstable under the photoirradiated conditions. Since the replacement of the phenylseleno group of **5** with the phenylthio group was relatively slow, the photoinduced reaction of **1k** with an equimolar mixture of (PhS)_2_ and (PhSe)_2_ was conducted at 25 °C upon irradiation with a 100 W xenon lamp for 50 h. As a result, the yield of **4** slightly improved (44% yield) (entry 5). In the absence of (PhSe)_2_, the desired dithiolation product **4** was obtained with only a 6% yield (entry 6).

We next investigated the photoinduced dithiolation of styrene **1j** in the presence of (PhSe)_2_. To suppress the thermal polymerization of styrene **1j** itself during the photoirradiation, the reaction vessels (NMR tubes) were immersed in water during light exposure to maintain the reaction temperature at 25 °C by measuring the water temperature with a thermometer. As shown in Table 2, the reaction of styrene **1j** (0.5 mmol) with (PhS)_2_ (1 mmol) in the presence of (PhSe)_2_ (30 mol%) in CDCl_3_ (0.5 mL) upon irradiation with a 100 W Xe lamp for 11 h led to the formation of dithiolation product **6a** and thioselenation product **7a** in 11% and 21% yields, respectively (entry 1). A high concentration of the substrates under irradiation with a 500 W Xe lamp improved the yield of **6a** to 30% (entry 2). When stoichiometric amounts of (PhSe)_2_ were used, the yield of **6a** increased to 35% without the formation of the thioselenation product **7a** (entry 3). Using up to 200 mol% (1.0 mmol) of (PhSe)_2_ produced **6a** in a similar yield and the thioselenation product **7a** in a 23% yield (entry 4). 

It was notable that (PhSe)_2_-assisted dithiolation of 4-trifluoromethylstyrene **1l** successfully proceeded to form the corresponding dithiolation product **6b** in 38% yield with good product selectivity (Equation (3)).

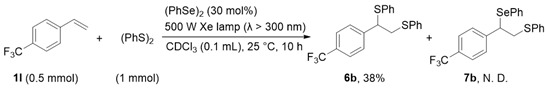
(3)

For vinylic ethers and styrenes, when the photoinduced reaction was examined using disulfides alone, a polymerization reaction occurred in preference to the desired dithiolation reaction. In contrast, it was found that the dithiolation reaction proceeded when diselenide was added to the system. These results strongly suggest that it is possible to replace the seleno group with a thio group for alkenes having active groups such as alkoxy and phenyl groups. 

In sharp contrast to the activated alkenes such as vinylic ethers and styrenes, nonactivated alkenes barely underwent photoinduced dithiolation using a (PhS)_2_-(PhSe)_2_ binary system. This is most probably due to the relatively strong bond energy of the C–Se bond. The next possibility for preferential dithiolation of such simple alkenes is to utilize the C–Te bond, which has a binding energy of 8 kcal/mol lower than the C–Se bond. Thus, we next focused on the utilization of the thiotelluration reaction system for the catalytic dithiolation of the unsaturated compounds. In fact, we previously reported two dithiolation reactions using the thiotelluration reaction system. One is the regioselective dithiolation of allenes in the presence of stoichiometric or catalytic amounts of ditelluride (Equation (4)) [28]. This dithiolation is considered to have proceeded successfully because allenes have an accumulated double bond and are highly reactive unsaturated bonds. Another example is the cycloadditive dithiolation of *o*-isocyanostyrene derivatives in the presence of stoichiometric amounts of ditelluride, in which the dithiolation reaction was successfully carried out by incorporating radical cyclization (Equation (5)) [29]. However, a radical dithiolation reaction to simple alkenes in the presence of ditelluride has not yet been successful.

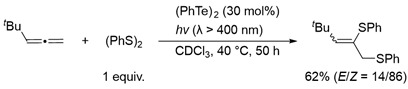
(4)
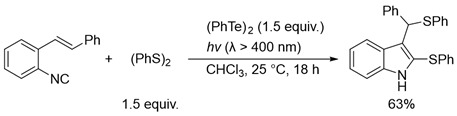
(5)

In our previous work, photoinduced thiotelluration proceeds in alkynes and in special alkenes such as norbornene **1h** [34]. Thus, we examined the photoinduced dithiolation of norbornene **1h** using a (PhS)_2_-(PhTe)_2_ system. In the presence of (PhTe)_2_ (30 mol%), the thiolation of norbornene **1h** proceeded to give the corresponding bisthiolated product **8** and the thiotelluration product **9** in 2% and 30% yields, respectively (entry 1, Table 3). The photoinduced reaction of **1h** and (PhS)_2_ with stoichiometric amounts of (PhTe)_2_ improved the yield of the thiotelluration product **9** (entry 3). Prolonging the irradiation time with 30 mol% of (PhTe)_2_ improved the yield of **8** (10%) (entry 4). It was suggested that it is not impossible to replace the C–Te bond in **9** with the C–S bond, although optimization of the reaction conditions is necessary.

The experimental results shown in Table 1, Table 2 and Table 3 suggest that not only the weakness of the carbon–chalcogen bond but also the stability of the carbon radicals generated in the system by the homolytic cleavage of the carbon–chalcogen bond are important in promoting the replacement of the seleno and telluro groups of the products by the thio group. 

Considering the weakness of the carbon–heteroatom bond and the stability of the generated radicals, we further investigated in detail the influence of the substituents adjacent to the carbon radicals on the radical substitution reaction. In the case of alkenes, the reaction proceeds by 1,2-addition, whereas the reaction of isocyanides bearing a C–N unsaturated bond proceeds by 1,1-addition. Therefore, the heteroatom radical attacks the isocyanide to form a radical on the carbon of the C=N bond, which is affected by the heteroatom group and the C–N double bond. Using isocyanide as a substrate, we investigated the photoinduced addition reaction and found that, interestingly, the catalytic dithiolation reaction with (PhS)_2_ occurs in the presence of (PhSe)_2_.

As shown in Table 4, the reaction of cyclohexyl isocyanide **10a** (0.25 mmol) with (PhS)_2_ (2.0 equiv.) in the presence of (PhSe)_2_ (30 mol%) under light afforded the 1,1-addition product **11a** in good yield (entry 1). High concentration of the starting materials led to the corresponding dithiolation product **11a** in up to 81% yield (entry 3). It is noteworthy that the thioselenation adduct **12a** was obtained in trace yields under the conditions of entries 1–3; therefore, the transformation of the in situ-generated **12a** to the dithiolation adduct **11a** might proceed very smoothly under these conditions. In the absence of (PhSe)_2_, the yield of **11a** dramatically decreased, indicating that the presence of catalytic amounts of (PhSe)_2_ was essential for the efficient transformation of **10a** to **11a** (entry 4). 

**Scheme 5:**
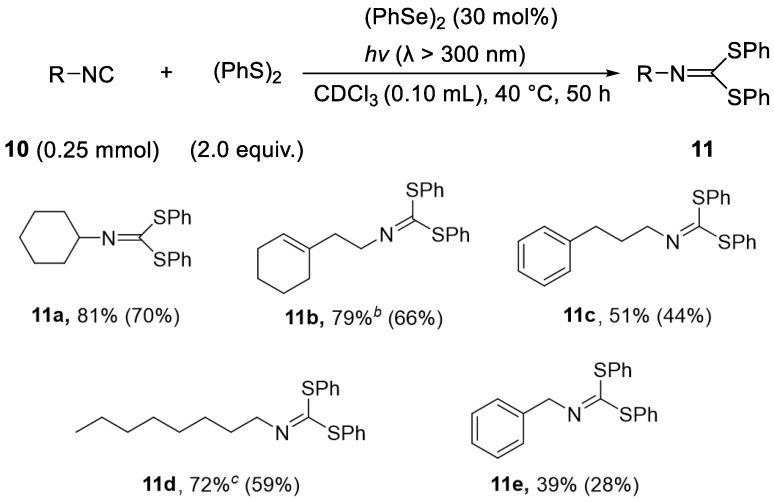
Scope and limitations of the (PhSe)_2_-catalyzed dithiolation of isocyanides *^a^*. *^a^* Yields were determined by ^1^H NMR spectroscopy (isolated yields). *^b^* Reaction time: 23 h. *^c^* The reaction was conducted without the solvent.

Scheme 5 shows the scope and limitations of the (PhSe)_2_-catalyzed dithiolation of isocyanides. Various isocyanide derivatives could be tolerated by the catalytic dithiolation, and the corresponding adducts **11a**–**11e** were obtained in moderate to good yields with excellent product selectivity.

As described above, in the addition to isocyanides, the C–Se bond generated by thioselenation could be converted to a C–S bond more efficiently than in the addition of alkenes. In other words, in the thioselenation of isocyanides, an addition product with an imino group (PhS–C(=N–R)–SePh) is formed, and the addition of a thiyl radical to this C=N bond and the subsequent elimination of the seleno radical can efficiently replace the seleno group of the thioselenation product with a thio group; thus, catalytic dithiolation is considered to proceed well.

## 3. Materials and Methods

### 3.1. General Information

Unless otherwise stated, all starting materials were purchased from commercial sources and used without further purification. Isocyanide derivatives **10b**–**10e** were prepared according to the previously reported procedures [39]. All solvents were distilled and degassed with argon before use. ^1^H and ^13^C{^1^H} NMR spectra were recorded in CDCl_3_ using a Bruker BioSpin Ascend 400 spectrometer (Tokyo, Japan) at 400 and 100 MHz, respectively, with Me_4_Si as the internal standard. High-resolution mass spectra were obtained on the JEOL JMS-700 Mstation (Tokyo, Japan) in the analytical section of the Nanotechnology Platform Program of the Nara Institute of Science and Technology (NAIST). The characterization data of compounds are shown as follows (^1^H and ^13^C{^1^H} NMR spectra are included in the Supplementary Materials).

### 3.2. (PhSe)_2_-Assisted Dithiolation of 2,3-Dihydrofuran 1k under Photoirradiation

In a sealed Pyrex NMR tube under an argon atmosphere, 2,3-Dihydrofuran **1k** (1.0 mmol), (PhS)_2_ (1.0 mmol), and (PhSe)_2_ (1.0 mmol) were placed, and the mixture was irradiated with a tungsten lamp (500 W) at a distance of 5 cm for 50 h at 40 °C. After the reaction, the resulting mixture was transferred to a flask, the solvent was removed under reduced pressure, and the residue was analyzed by NMR spectroscopy using 1,3,5-trioxane as the internal standard. The production of **4** was determined and characterized from the reported ^1^H and ^13^C{^1^H} NMR data (Table 1) [40].

### 3.3. (PhSe)_2_-Assisted Dithiolation of Styrene 1j under Photoirradiation

Styrene **1j** (0.5 mmol), (PhS)_2_ (1.0 mmol), and (PhSe)_2_ (30 mol%) in degassed CDCl_3_ (0.1 mL) were placed in a sealed Pyrex NMR tube under an argon atmosphere, and the mixture was irradiated with a xenon lamp (500 W) at a distance of 5 cm for 50 h at 25 °C. To suppress the thermal polymerization of styrene **1j** itself during the photoirradiation, the reaction vessels (NMR tubes) were immersed in water during light exposure to maintain the reaction temperature at 25 °C by measuring the water temperature with a thermometer. After the reaction, the resulting mixture was transferred to a flask, the solvent was removed under reduced pressure, and the residue was analyzed by NMR spectroscopy using 1,3,5-trioxane as the internal standard. The production of **6a** was determined and characterized from the reported ^1^H and ^13^C{^1^H} NMR data (Table 2) [41].

### 3.4. (PhTe)_2_-Assisted Dithiolation of Norbornene 1h under Photoirradiation

Norbornene **1h 1j** (0.5 mmol), (PhS)_2_ (2.0 mmol), and (PhTe)_2_ (30 mol%) in degassed CDCl_3_ (0.05 mL) were placed in a sealed Pyrex NMR tube under an argon atmosphere, and the mixture was irradiated with a tungsten lamp (500 W) at a distance of 5 cm for 50 h. After the reaction, the resulting mixture was transferred to a flask, the solvent was removed under reduced pressure, and the residue was analyzed by NMR spectroscopy using 1,3,5-trioxane as the internal standard. The production of **8** and **9** was determined and characterized from the reported ^1^H and ^13^C{^1^H} NMR data (Table 3) [34].

### 3.5. General Procedure for the (PhSe)_2_-Catalyzed Dithiolation of Isocyanides

Isocyanide **10** (0.25 mmol), (PhS)_2_ (2.0 mmol), and (PhSe)_2_ (30 mol%) in degassed CDCl_3_ (0.1 mL) were placed in a sealed Pyrex NMR tube under an argon atmosphere, and the mixture was irradiated with a tungsten lamp (500 W) at a distance of 5 cm for 50 h at 40 °C. After the reaction, the resulting mixture was transferred to a flask, and the solvent was removed under reduced pressure. Finally, the residue was purified by preparative thin-layer chromatography (eluent: *iso*-hexane/Et_2_O) to obtain the corresponding dithiolation product **11** (Scheme 5).

Diphenyl cyclohexylcarbonimidodithioate (**11a**) [CAS no. 924622-07-1] [31]. White solid, 57.5 mg, 70%; ^1^H NMR (400 MHz, CDCl_3_) δ 7.51 (d, *J* = 6.8 Hz, 2H), 7.35-7.26 (m, 8H), 3.81-3.75 (m, 1H), 1.68-1.65 (m, 4H), 1.56-1.54 (m, 2H), 1.43-1.18 (m, 4H); ^13^C{^1^H} NMR (100 MHz, CDCl_3_): δ 151.9, 135.1, 134.0, 131.6, 130.5, 129.0 (two carbons were overlapped), 128.4, 128.2, 62.6, 33.0, 25.8, 24.2.

Diphenyl (2-(cyclohex-1-en-1-yl)ethyl)carbonimidodithioate (**11b**) [CAS no. 924622-08-2] [31]. Yellow oil, 63.3 mg, 66%; ^1^H NMR (400 MHz, CDCl_3_) δ 7.56-7.54 (m, 2H), 7.39-7.34 (m, 5H), 7.28-7.26 (m, 3H), 5.37-5.37 (m, 1H), 3.60 (t, *J* = 7.0 Hz, 2H), 2.18 (t, *J* = 7.1 Hz, 2H), 1.98-1.97 (m, 2H), 1.88-1.88 (m, 2H), 1.62-1.51 (m, 4H); ^13^C{^1^H} NMR (100 MHz, CDCl_3_): δ 154.6, 135.8, 135.1, 134.5, 131.0, 130.1, 129.2, 129.1, 128.6, 128.5, 122.6, 53.3, 38.6, 28.5, 25.4, 23.1, 22.5.

Diphenyl (3-phenylpropyl)carbonimidodithioate (**11c**). Yellow oil, 38.4 mg, 44%; ^1^H NMR (400 MHz, CDCl_3_) δ 7.58-7.56 (m, 2H), 7.43-7.35 (m, 5H), 7.31-7.30 (m, 3H), 7.27-7.24 (m, 2H), 7.18-7.14 (m, 1H), 7.10-7.08 (m, 2H), 3.48 (t, J = 6.6 Hz, 2H), 2.56 (t, J = 7.8 Hz, 2H), 1.87-1.80 (m, 2H); ^13^C{^1^H} NMR (100 MHz, CDCl_3_): δ 155.0, 142.3, 135.4, 134.8, 131.0, 129.8, 129.3, 129.1, 128.7, 128.6 (two carbons were overlapped), 128.3, 125.7, 53.2, 33.5, 32.3; HRMS (CI) calcd for C_22_H_22_NS_2_ [M+H]^+^: 364.1194, found: 363.1194.

Diphenyl octylcarbonimidodithioate (**11d**). Colorless oil, 57.2 mg, 59%; ^1^H NMR (400 MHz, CDCl_3_) δ 7.55-7.54 (m, 2H), 7.39-7.34 (m, 5H), 7.30-7.27 (m, 3H), 3.49 (t, *J* = 6.9 Hz, 2H), 1.56-1.51 (m, 2H), 1.34-1.25 (m, 10H), 0.90-0.87 (m, 3H); ^13^C{^1^H} NMR (100 MHz, CDCl_3_): δ 154.3, 135.2, 134.4, 131.1, 130.1, 129.2, 129.1, 128.6, 128.5, 54.3, 31.9, 30.4, 29.3 (two carbons were overlapped), 27.3, 22.7, 14.2; HRMS (CI) calcd for C_21_H_28_NS_2_ [M+H]^+^: 358.1663, found: 358.1670.

Diphenyl benzylcarbonimidodithioate (**11e**) [CAS no. 924622-05-9] [31]. White solid, 25.4 mg, 28%; ^1^H NMR (400 MHz, CDCl_3_) δ 7.61 (dd, *J* = 7.8, 1.5 Hz, 2H), 7.43-7.37 (m, 5H), 7.33-7.30 (m, 3H), 7.27-7.23 (m, 2H), 7.20-7.15 (m, 3H), 4.72 (s, 2H); ^13^C{^1^H} NMR (100 MHz, CDCl_3_): δ 157.0, 139.6, 135.6, 135.1, 130.7, 129.6, 129.4, 129.2, 128.8, 128.7, 128.2, 127.2, 126.5, 57.0.

## 4. Conclusions

In this study, we investigated the multiple introductions of chalcogen functional groups into various unsaturated bonds under radical conditions and examined in detail whether the selenium and tellurium functional groups of the thioselenation or thiotelluration products can be replaced by a sulfur functional group under radical reaction conditions. For simple alkenes without an active substituent, it was difficult to replace the C–Se or C–Te bond of the thioselenation or thiotelluration products with a C–S bond, but for the activated alkenes such as vinylic ethers and styrenes, it was found that it was possible to replace the seleno group with a thio group. In addition, isocyanides were successfully bisthiolated by photoirradiation in the presence of catalytic (PhSe)_2_ to afford the corresponding adducts in moderate to high yields with excellent product selectivity. Although the establishment of a “third” catalytic radical reaction, in which a typical element radical catalyzes the radical reactions of a homologous heteroatom compound, is still a challenge, the relative reactivities of unsaturated compounds demonstrated in this study are a new milestone in considering the substitution of the carbon–chalcogen bond by sulfur functional groups under radical conditions. We believe that this research on the development of a “third” catalytic radical reaction will lead to a new approach for the precise introduction of heteroatom-centered functional groups. 

## Figures and Tables

**Table 1:** (PhSe)_2_-assisted dithiolation of 2,3-dihydrofuran **1k** under photoirradiation.

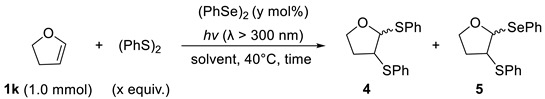						
Entry	(PhS)_2_ (Equiv.)	(PhSe)_2_ (mol%)	Solvent (mL)	Time (h)	Yield (%) *^a^*	
					4 [*trans/cis*]	5
1	0.5	30	neat	20	29 [79:21]	4
2	0.5	30	CDCl_3_ (0.10)	20	20 [75:25]	2
3	0.5	30	neat	100	8	N. D.
4	1.0	100	neat	20	41 [93:7]	3
5 *^b^*	1.0	100	neat	50	44 [89:11]	trace
6	1.0	-	neat	20	6 [83:17]	N. D.

*^a^* Yields were determined by ^1^H NMR spectroscopy. *^b^* 100 W xenon lamp was used, and the reaction was conducted at 25 °C.

**Table 2:** Attempted dithiolation of styrene **1j** assisted by (PhSe)_2_.

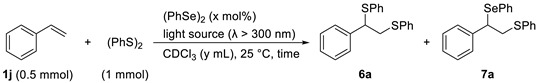							
Entry	(PhSe)_2_ (mol%)	Light Source	CDCl_3_ (mL)	Time (h)	Yield (%) *^a^*		Recovery 1j (%) *^a^*
					6a	7a	
1	30	100 W Xe lamp	0.5	11	11	21	33
2	30	500 W Xe lamp	0.1	10	30	N. D.	N. D.
3	100	500 W Xe lamp	0.1	10	35	N. D.	4
4	200	500 W Xe lamp	0.1	10	39	23	13

*^a^* Yields were determined by ^1^H NMR spectroscopy.

**Table 3:** Attempted dithiolation of norbornene **1h** assisted by (PhTe)_2_ under photoirradiation.

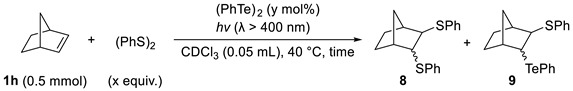					
Entry	(PhS)_2_ (Equiv.)	(PhTe)_2_ (mol%)	Time (h)	Yield (%) *^a^*	
				8	9 [*endo*/*exo*]
1 *^b^*	2.0	30	13	2	30 [23:77]
2	1.0	30	26	4	40 [20:80]
3	2.0	100	26	3	60 [25:75]
4	2.0	30	50	10	39 [37:63]
5 *^c^*	2.0	30	26	7	40 [21:79]

*^a^* Yields were determined by ^1^H NMR spectroscopy. *^b^* Norbornene **1h** (0.25 mmol) and CDCl_3_ (0.25 mL) were used. *^c^* Norbornene **1h** (1.0 mmol) was used.

**Table 4:** Optimization of the reaction conditions for (PhSe)_2_-catalyzed dithiolation of cyclohexyl isocyanide **10a**.

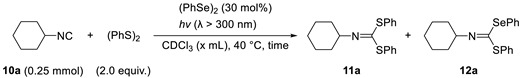					
Entry	CDCl_3_ (mL)	Time (h)	Yield (%) *^a^*		Recovery of 10a (%) *^a^*
			11a	12a	
1	0.50	50	45	trace	50
2	0.10	24	61	trace	31
3	0.10	50	81 (70)	trace	13
4 *^b^*	0.10	50	11	N. D.	85

*^a^* Yields were determined by ^1^H NMR spectroscopy (isolated yield). *^b^* In the absence of (PhSe)_2_.

## Data Availability

The data presented in this study are available in the article and in its Supplementary Materials.

## Supplementary Materials

The following supporting information can be downloaded at: https://www.mdpi.com/article/10.3390/molecules28062450/s1. Copies of ^1^H and ^13^C{^1^H} NMR spectra.

## Sample Availability

Samples of the compounds are not available from the authors.
