# Supplementary material for: Diphenyl Diselenide-Assisted Radical Addition Reaction of Diphenyl Disulfide to Unsaturated Bonds upon Photoirradiation
Source: Molecules. 2023 Mar 7;28(6):2450. doi: 10.3390/molecules28062450 (PMC10059204; doi:10.3390/molecules28062450)
Supplement: Supplementary file 1 [file molecules-28-02450-s001.zip › molecules-2223907-supplementary.pdf]

molecules

MDPI

Supplementary Materials

Diphenyl Diselenide-Assisted Radical Addition Reaction of Diphenyl Disulfide to Unsaturated Bonds upon Photoirradiation

Yuki Yamamoto[1], Qiqi Chen[2], and Akiya Ogawa[1,2,] *

1. Department of Applied Chemistry, Graduate School of Engineering, Osaka Prefecture University, Osaka 599-8531, Japan; syb02137@st.osakafu-u.ac.jp (Y.Y.)
2. Department of Applied Chemistry, Graduate School of Engineering, Osaka Metropolitan University, Osaka 599-8531, Japan; sc22033n@st.omu.ac.jp (Q. C.)

* Correspondence: ogawa@omu.ac.jp

Contents

Copies of ^{1}H and ^{13}C{^{1}H} NMR spectra ···2–6

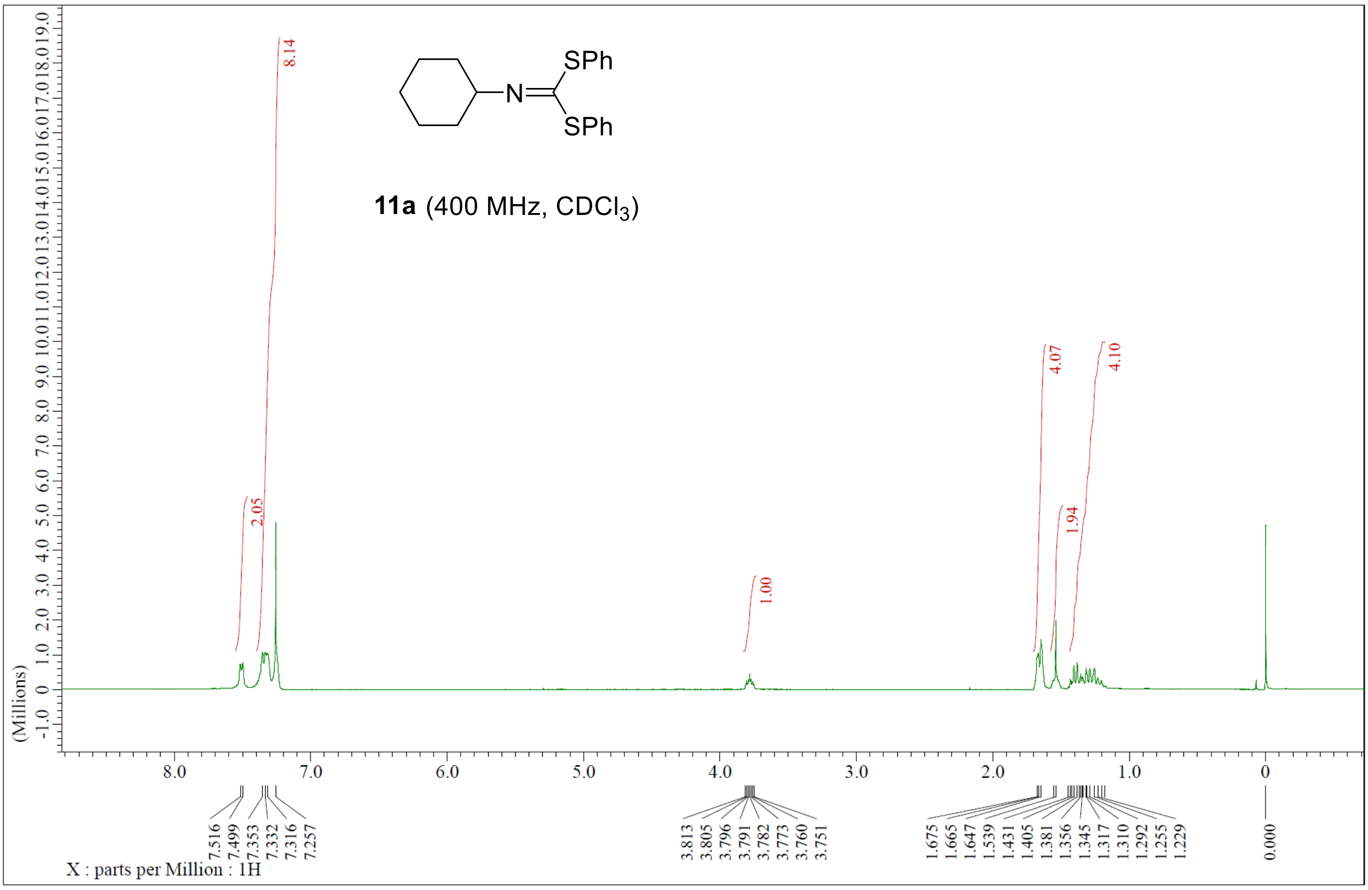
SPh
N
SPh
11a (400 MHz, $CDCl_3$)
8.14
2.05
1.00
4.07
1.94
4.10
(Millions)
7.516
7.499
7.353
7.332
7.316
7.257
3.813
3.805
3.796
3.791
3.782
3.773
3.760
3.751
1.675
1.665
1.647
1.539
1.431
1.405
1.381
1.356
1.345
1.317
1.310
1.292
1.255
1.229
0.000
X : parts per Million : 1H

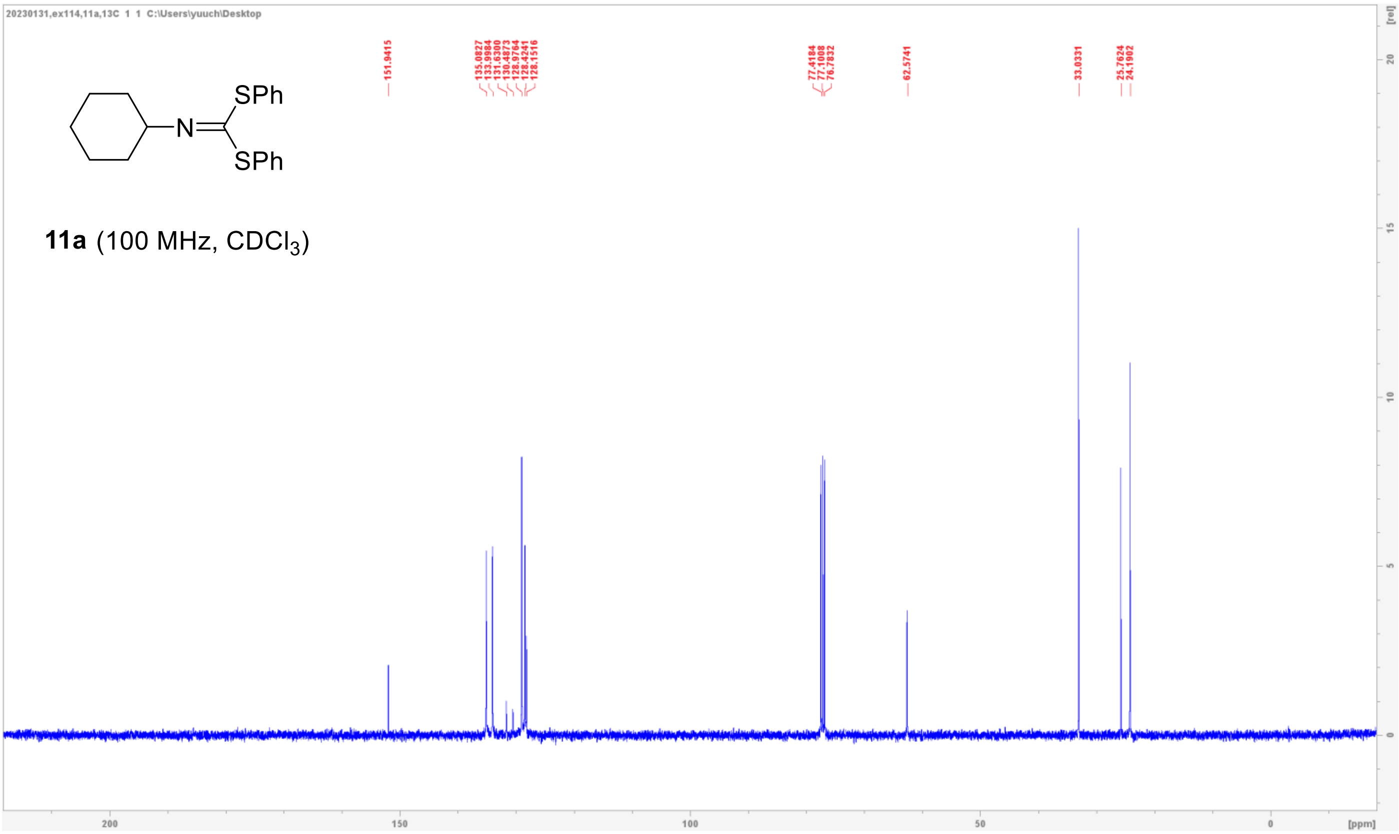
20230131,ex114,11a,13C 1 1 C:\Users\yuuch\Desktop
151.9415
135.0827
133.9984
131.6300
130.4873
128.9764
128.4241
128.1516
77.4184
77.1008
76.7832
62.5741
33.0331
25.7624
24.1902
SPh
N
SPh
11a (100 MHz, $CDCl_3$)
[ppm]
[rel]

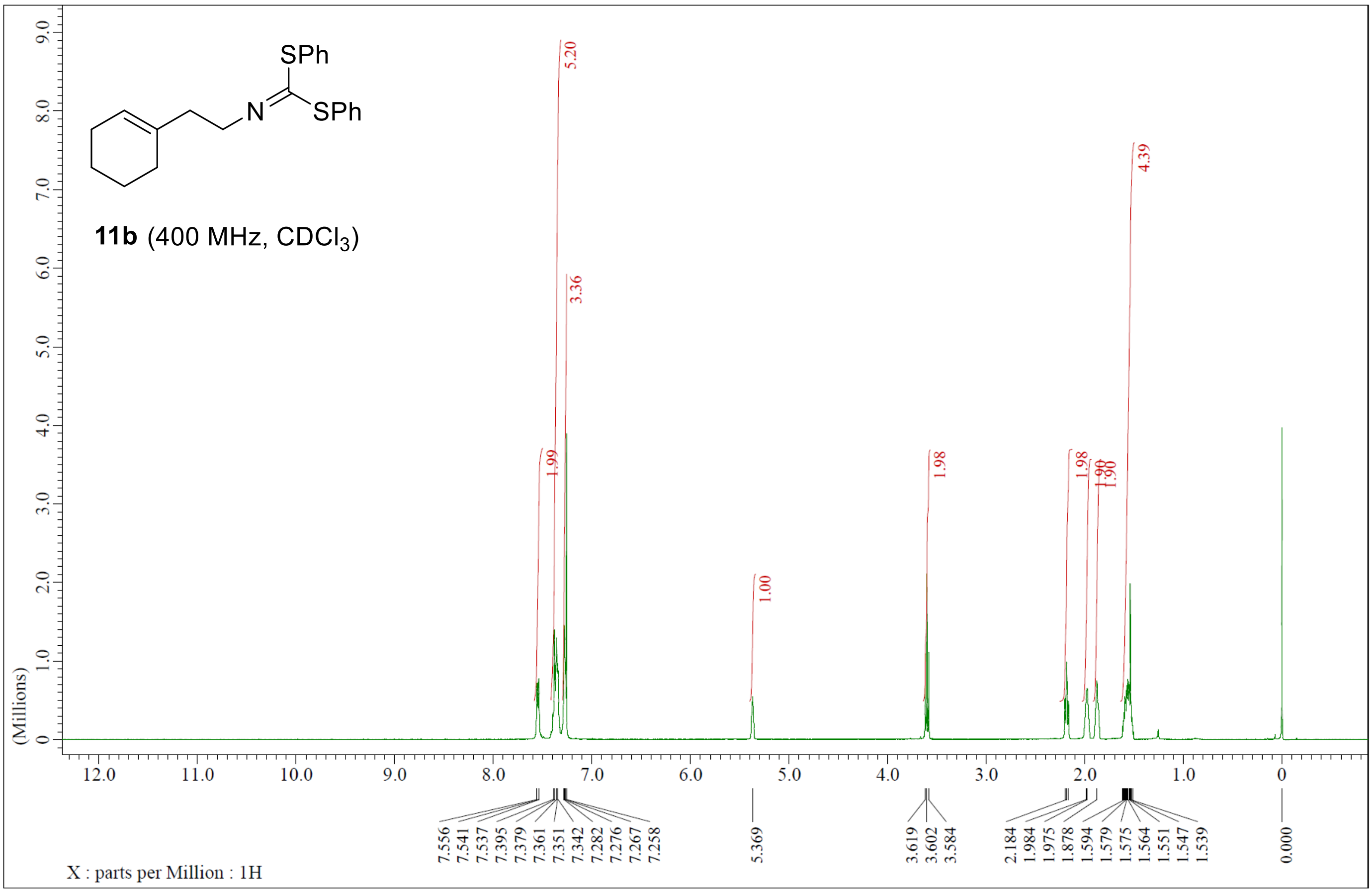
SPh
N
SPh
11b (400 MHz, $CDCl_3$)
(Millions)
X : parts per Million : 1H
7.556
7.541
7.537
7.395
7.379
7.361
7.351
7.342
7.282
7.276
7.267
7.258
5.369
3.619
3.602
3.584
2.184
1.984
1.975
1.878
1.594
1.579
1.575
1.564
1.551
1.547
1.539
0.000
1.99
5.20
3.36
1.00
1.98
1.98
1.90
1.90
4.39

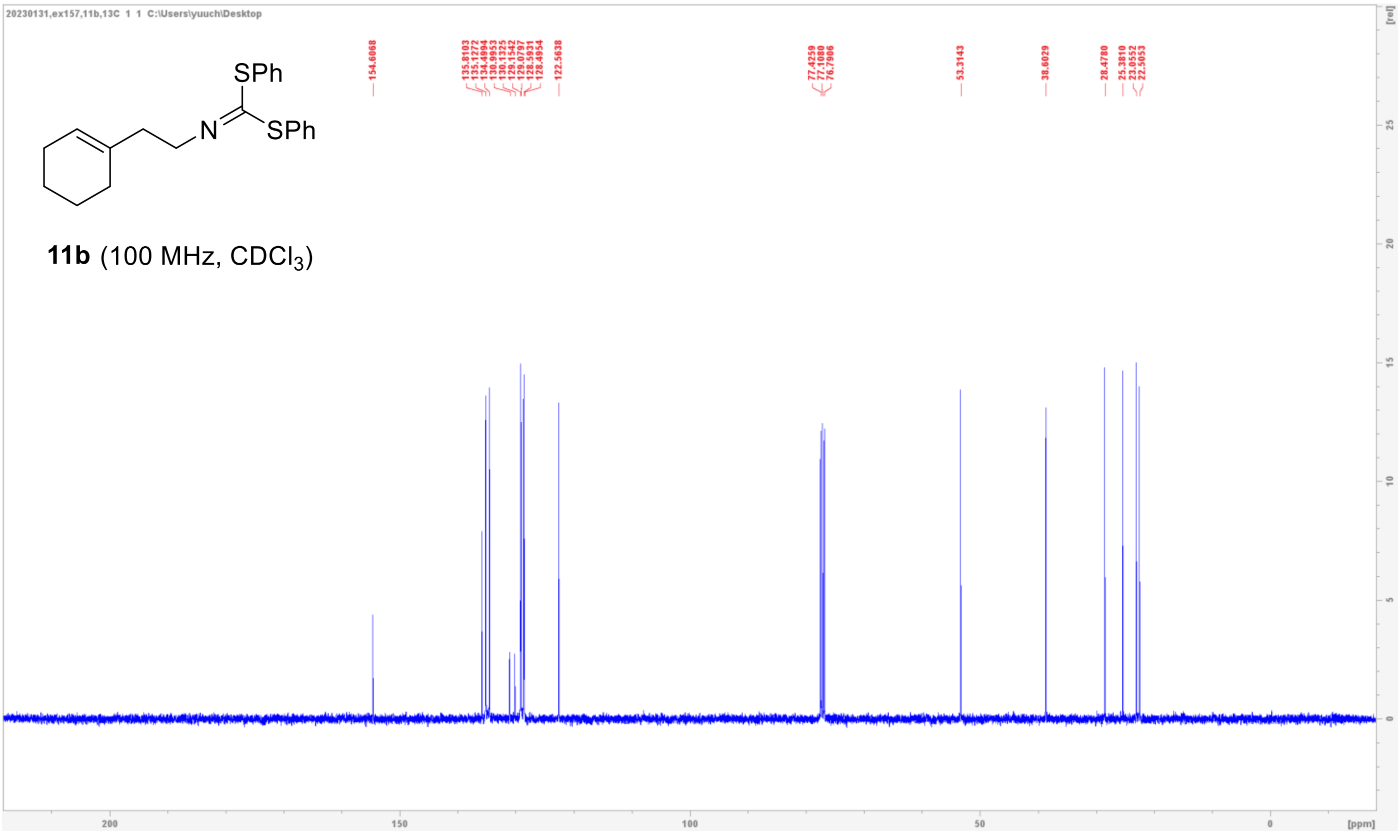
SPh
N
SPh
11b (100 MHz, $CDCl_3$)
[ppm]

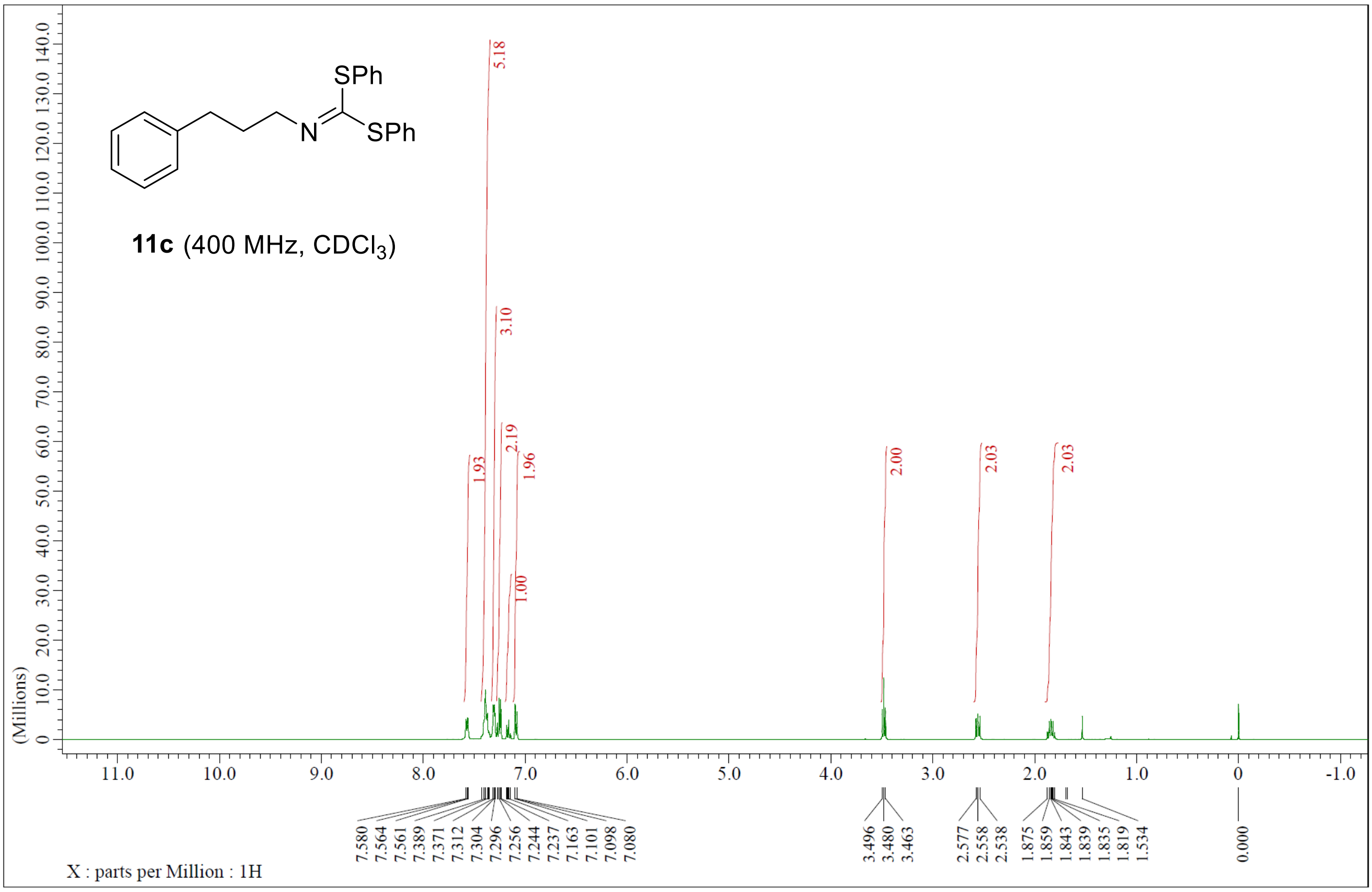
SPh
N
SPh
11c (400 MHz, CDCl3)
(Millions)
X : parts per Million : 1H
5.18
3.10
2.19
1.93
1.96
1.00
2.00
2.03
2.03
7.580
7.564
7.561
7.389
7.371
7.312
7.304
7.296
7.256
7.244
7.237
7.163
7.101
7.098
7.080
3.496
3.480
3.463
2.577
2.558
2.538
1.875
1.859
1.843
1.839
1.835
1.819
1.534
0.000

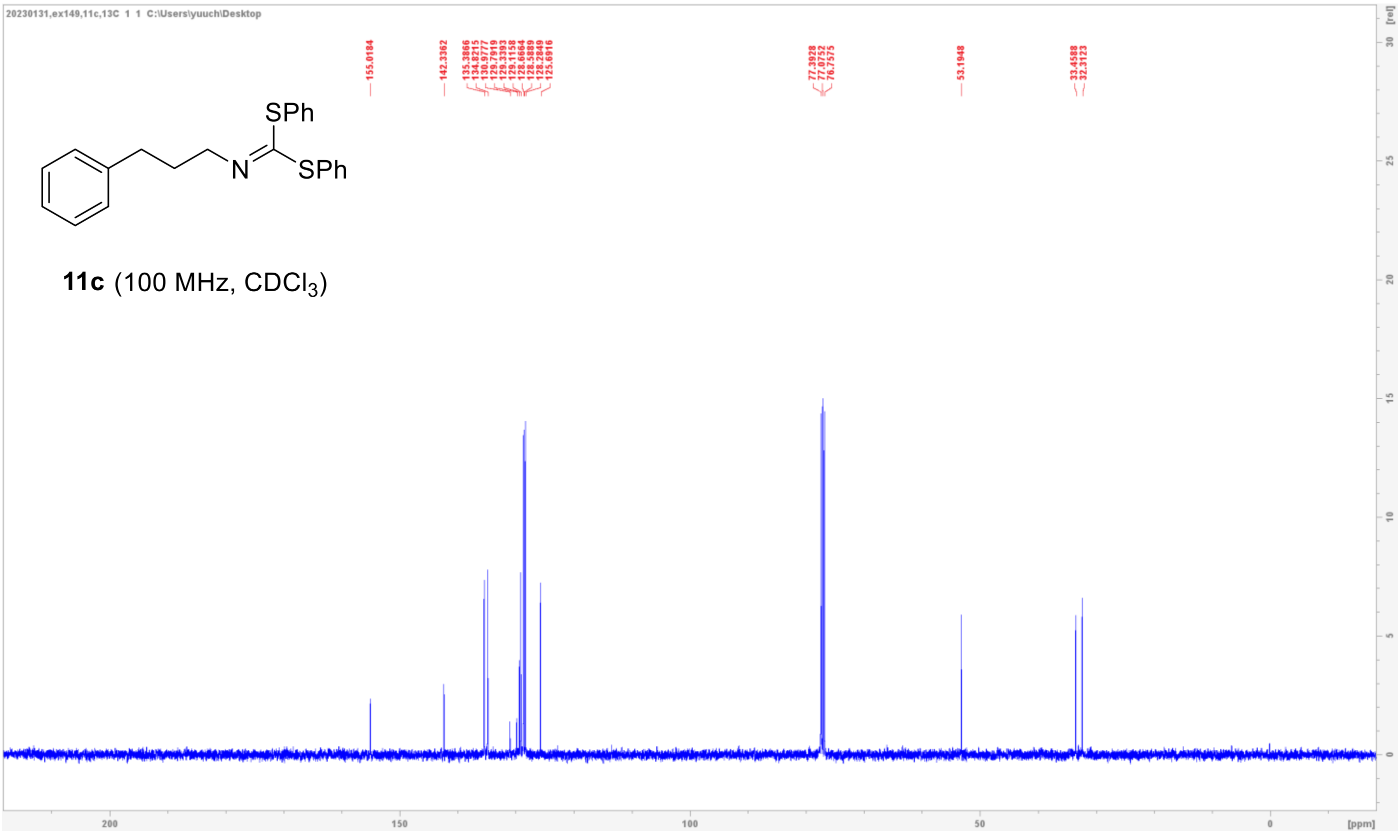
20230131,ex149,11c,13C 1 1 C:\Users\yuuch\Desktop
SPh
N
SPh
11c (100 MHz, CDCl3)
155.0184
142.3362
135.3866
134.8215
130.9777
129.7919
129.3393
129.1158
128.6664
128.5889
128.2849
125.6916
77.3928
77.0752
76.7575
53.1948
33.4588
32.3123
[ppm]
[rel]

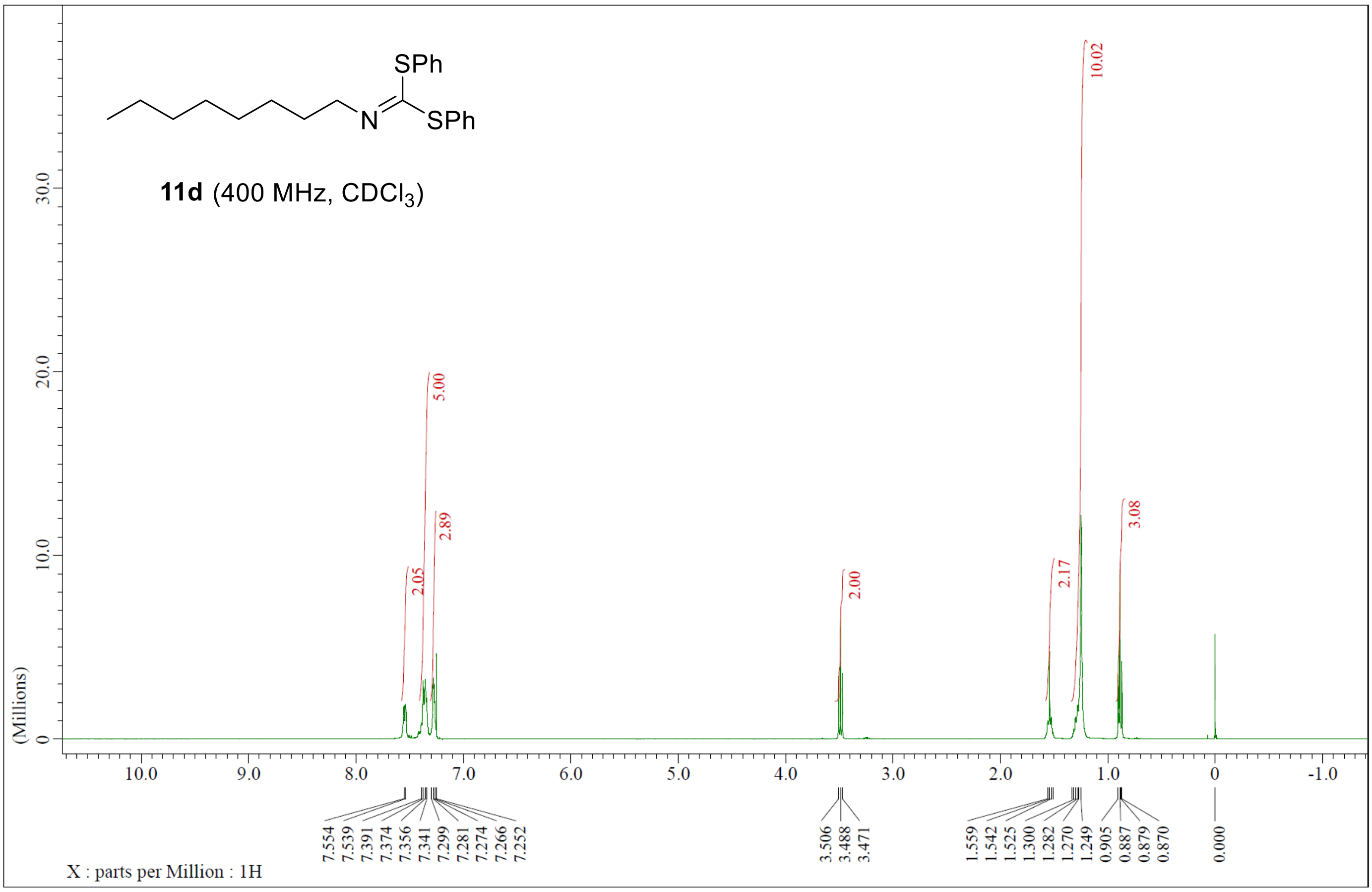
SPh
N
SPh
11d (400 MHz, CDCl3)
(Millions)
X : parts per Million : 1H
7.554
7.539
7.391
7.374
7.356
7.341
7.299
7.281
7.274
7.266
7.252
3.506
3.488
3.471
1.559
1.542
1.525
1.300
1.282
1.270
1.249
0.905
0.887
0.879
0.870
0.000
2.05
5.00
2.89
2.00
2.17
10.02
3.08

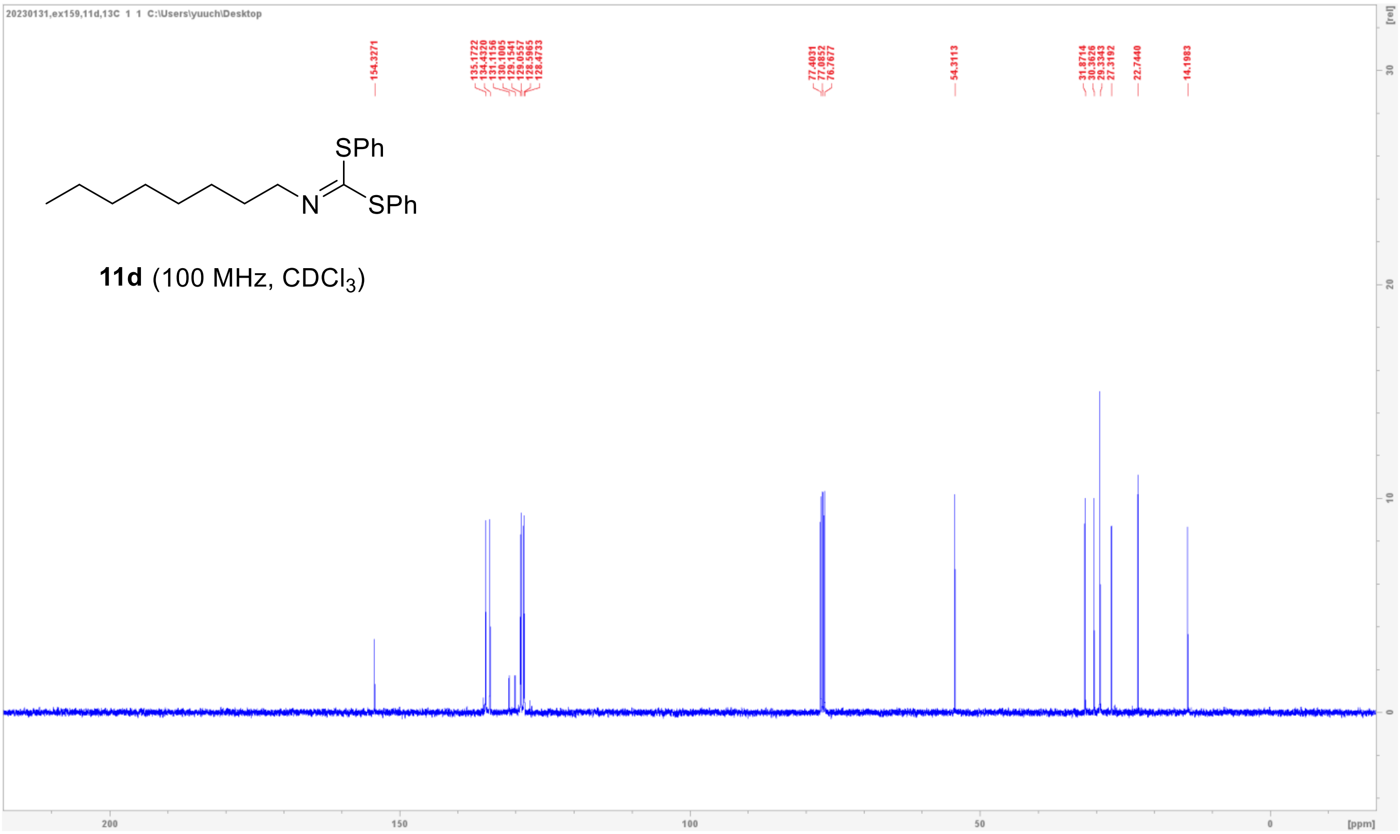
SPh
N
SPh
11d (100 MHz, CDCl3)
154.3271
77.4031
54.3113
22.7440
14.1983
[ppm]

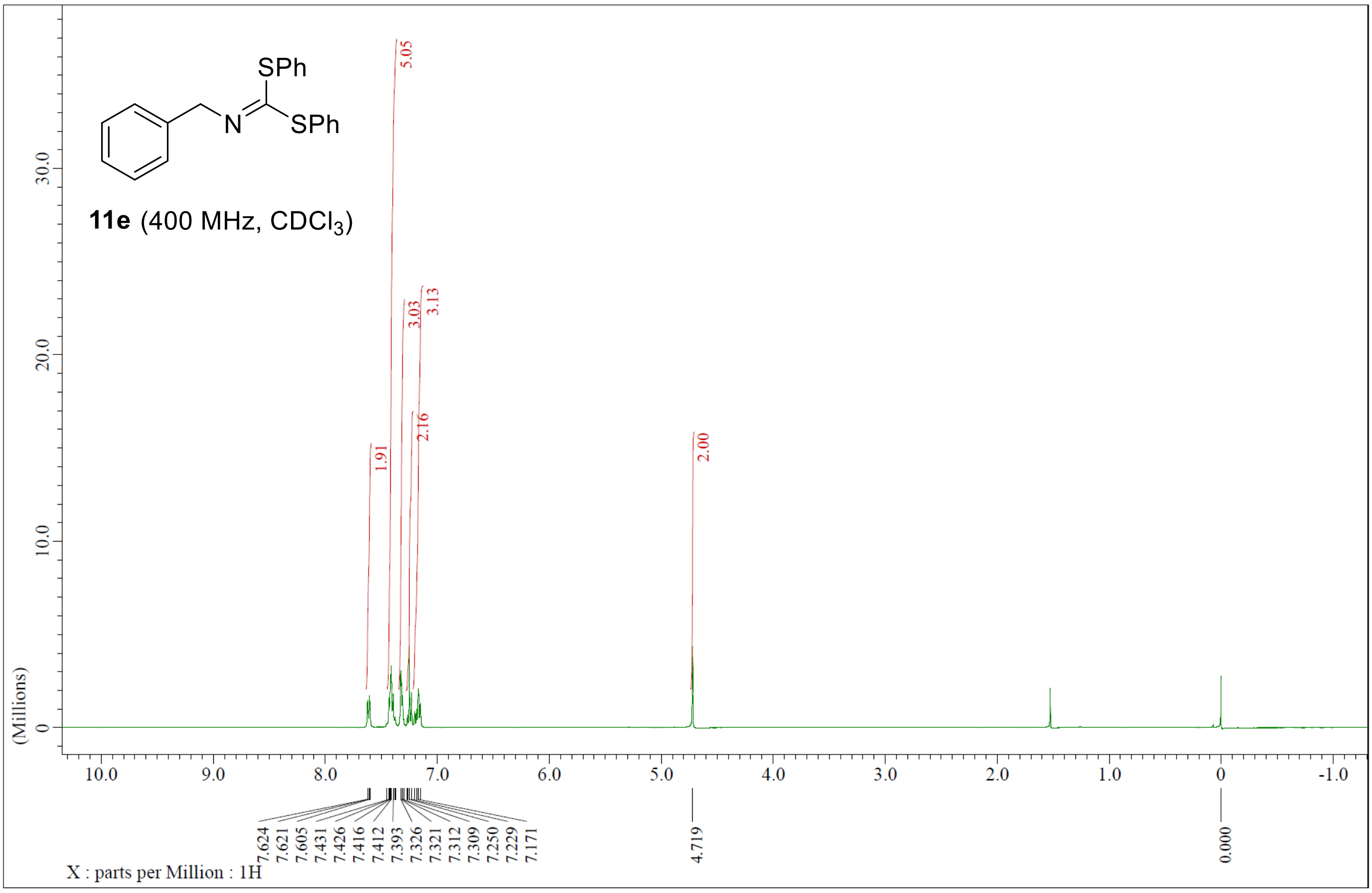
SPh
N
SPh
11e (400 MHz, CDCl3)
(Millions)
0
10.0
20.0
30.0
1.91
5.05
3.03
3.13
2.16
2.00
10.0
9.0
8.0
7.0
6.0
5.0
4.0
3.0
2.0
1.0
0
-1.0
7.624
7.621
7.605
7.431
7.426
7.416
7.412
7.393
7.326
7.321
7.312
7.309
7.250
7.229
7.171
4.719
0.000
X : parts per Million : 1H

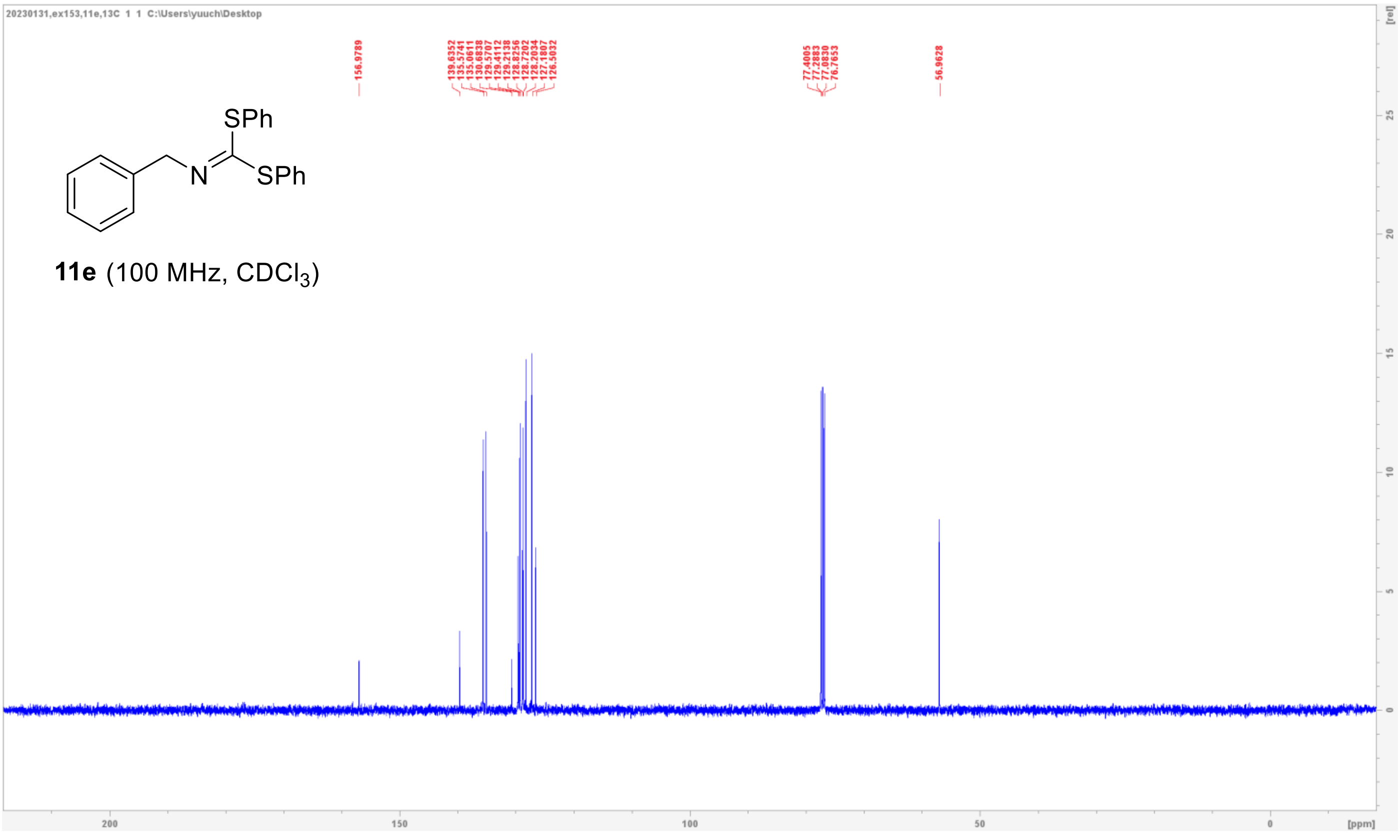
SPh
N
SPh
11e (100 MHz, CDCl3)
200
150
100
50
0
[ppm]
